# Supplementary material for: “We’re here to help them if they want to come”: A qualitative exploration of hospital staff perceptions and experiences with outpatient non-attendance
Source: PLoS One. 2025 Jun 9;20(6):e0311059. doi: 10.1371/journal.pone.0311059 (PMC12148132; doi:10.1371/journal.pone.0311059)
Supplement: S1 Checklist — (DOCX) [file pone.0311059.s001.docx]

**Supplementary Material**

**Consolidated Criteria for Reporting Qualitative Studies (COREQ) Checklist**

*Table S1. Completed COREQ 32-item checklist for transparent and complete reporting of methods*

| **COREQ checklist** | **Location where item is reported** |
| --- | --- |
| **Domain 1: Research team and reflexivity** | |
| *Personal Characteristics* |  |
| 1. Interviewer/facilitator | Methods - Data collection |
| 2. Credentials | Methods - Data collection |
| 3. Occupation | Methods - Data collection |
| 4. Gender | Methods - Data collection |
| 5. Experience and training | Methods - Data collection |
| *Relationship with participants* |  |
| 6. Relationship established | Methods - Data collection |
| 7. Participant knowledge of the interviewer | Methods - Data collection |
| 8. Interviewer characteristics | Methods - Data collection |
| **Domain 2: study design** |  |
| *Theoretical framework* |  |
| 9. Methodological orientation and theory | Methods - Study design & methodological approach |
| *Participant selection* |  |
| 10. Sampling | Methods - Study participants and recruitment |
| 11. Method of approach | Methods - Study participants and recruitment |
| 12. Sample size | Methods - Study participants and recruitment |
| 13. Non-participation | Results - Participant characteristics |
| *Setting* |  |
| 14. Setting of data collection | Methods - Data collection |
| 15. Presence of non-participants | Methods - Data collection |
| 16. Description of sample | Methods - Study participants and recruitment |
| *Data collection* |  |
| 17. Interview guide | Methods - Data collection |
| 18. Repeat interviews | Methods - Data collection |
| 19. Audio/visual recording | Methods - Data collection |
| 20. Field notes | Methods - Data collection |
| 21. Duration | Methods - Data collection |
| 22. Data saturation | Methods - Data collection |
| 23. Transcripts returned | Methods - Data collection |
| **Domain 3: analysis and findings** |  |
| *Data analysis* |  |
| 24. Number of data coders | Methods - Data analysis |
| 25. Description of the coding tree | Methods - Data analysis |
| 26. Derivation of themes | Methods - Data analysis |
| 27. Software | Methods - Data analysis |
| 28. Participant checking | Methods - Data collection |
| *Reporting* |  |
| 29. Quotations presented | Results |
| 30. Data and findings consistent | Results |
| 31. Clarity of major themes | Results |
| 32. Clarity of minor themes | Results |
