# Supplementary material for: “We’re here to help them if they want to come”: A qualitative exploration of hospital staff perceptions and experiences with outpatient non-attendance
Source: PLoS One. 2025 Jun 9;20(6):e0311059. doi: 10.1371/journal.pone.0311059 (PMC12148132; doi:10.1371/journal.pone.0311059)
Supplement: S2 File — (DOCX) [file pone.0311059.s002.docx]

**Supplementary Material**

**Interview Guide (semi-structured interviews / focus groups)**

**Interview aims**

- Explore healthcare professionals’ perspectives, beliefs, and opinions about the impact of patient non-attendance within a publicly-funded outpatient physiotherapy clinic context
- Explore perceived barriers and facilitators associated with the implementation of non-attendance mitigation strategies
- Identify health service staff generated solutions to address perceived barriers and enhance facilitators using a theory-informed approach

**How to use this guide**

*Development of the guide*

An initial discussion with key stakeholders was conducted to clearly define and explore the issue of outpatient non-attendance within the physiotherapy outpatient context, concordant with the phenomenological approach. These key stakeholders included a clinic director (n=1), physiotherapist clinic managers (n=2), and an administrative manager (n=1). The discussion centred on understanding relevant work processes, and latent protocols, as they related to the experience on non-attendance within their practice, so that the interview guide questions were informative and contextually-relevant to participating physiotherapy clinic staff. Topics were developed to explore participants’ perceived thoughts on the impacts of non-attendance, perceptions of the barriers and facilitators to patient attendance, and opinions about current or prospective non-attendance mitigation strategies.

*Use of the guide*

The interviewer should follow the questions and prompts from the section(s) of the guide. Within each section, remember:

1. It is not necessary for interviewers to ask questions in the proposed order, or ask all questions exactly as stated, or repeat a question if it has been answered in an earlier section
2. Should other relevant issues be raised, they will be explored in the relevant section, with guidance and prompting as required
3. Interviewers will ensure discussion progresses in a timely, yet informative manner

**Introduction**

- Introduce self and the study
- Explain how interview will unfold

**Interview questions**

**PARTICIPANT BACKGROUND (ROLE AT THE CLINIC)**

1. **Can you tell me a little bit about yourself and your current role at the clinic?**

Prompts:

*Tell me what it is you do at the clinic?*

*How many people do you work with?*

*Do you currently work part-time or on a full-time basis?*

**PERCEPTIONS AROUND NON-ATTENDANCE**

1. **From your perspective and clinical experience, has non-attendance happened with enough frequency (in your clinic) to be of concern?**

Prompts:

*Is it something you think we should be worried about in the health system?*

*Do you find that it impacts you (or your workflow)? If so, how has it impacted you (or your work)?*

*Do you find that it has an impact on patients? If so, how may it impact patients?*

1. **Let’s talk about the different types of non-attendance that could occur at your clinic.**

Prompts:

*No-show to scheduled appointments*

*Last minute cancellations*

*Not attending follow up appointments*

*Rescheduled and did not attend*

*Other types*

**BARRIERS AND FACILITATORS TO ATTENDANCE / PERCEPTIONS AROUND REASONS PATIENTS ATTEND AND DON’T ATTEND APPOINTMENTS**

1. **What are your thoughts on who might be more likely to attend / not-attend?**

Prompts:

*Why might that be the case?*

*What factors do you think contributes to attendance / non-attendance?*

**VIEWS ON CURRENT AND PROSPECTIVE SOLUTIONS FOR NON-ATTENDANCE**

1. **Could you tell me about the solutions or processes you have in place to mitigate non-attendance at your clinic?**

Prompts:

*What solutions are in place?*

*Do you find that these solutions are working well?*

*What hasn’t been working?*

*What characteristics of those solutions work well / don’t work well?*

*What are the challenges of using those solutions within your practice?*

1. **In your opinion, how would you describe some things that your clinic does to enable your patients to attend their appointments?**

Prompts:

*Reminders*

*Follow ups*

*Penalties for non-attendance*

*Other*

1. **What are some things that you would suggest to aid in reducing the impact of patient non-attendance at your clinic?**

Prompts:

*Are there any solutions you think could work well to reduce the impact of non-attendance?*

*Are there any solutions you think would not work well to reduce the impact of non-attendance?*

*What characteristics of those solutions do you think would work well / not work well?*

*What would be the challenges for using those solutions within your practice?*

**CONCLUDING QUESTIONS**

1. **If you had an unlimited budget, resources and time, what are some things that you would suggest to health care policy makers and other decision makers which could systematically reduce the impact of patient non-attendance within the health service?**

Prompts:

*Are there any solutions you think could work well to reduce the impact of non-attendance?*

*Why those solutions?*

1. **Is there anything else you wanted to add which we haven’t covered regarding non-attendance at your clinic or the health system at large?**

*Thank you very much for taking part in this interview.*

*End of guide*
